# Longitudinal engagement trajectories and risk of death among new ART starters in Zambia: A group-based multi-trajectory analysis

**DOI:** 10.1371/journal.pmed.1002959

**Published:** 2019-10-29

**Authors:** Aaloke Mody, Ingrid Eshun-Wilson, Kombatende Sikombe, Sheree R. Schwartz, Laura K. Beres, Sandra Simbeza, Njekwa Mukamba, Paul Somwe, Carolyn Bolton-Moore, Nancy Padian, Charles B. Holmes, Izukanji Sikazwe, Elvin H. Geng

**Affiliations:** 1 Division of HIV, ID and Global Medicine, University of California, San Francisco, Zuckerberg San Francisco General Hospital, San Francisco, California, United States of America; 2 Centre for Infectious Diseases Research in Zambia, Lusaka, Zambia; 3 Department of International Health, Bloomberg School of Public Health, Johns Hopkins University, Baltimore, Maryland, United States of America; 4 Division of Infectious Diseases, University of Alabama at Birmingham, Alabama, United States of America; 5 Division of Epidemiology, University of California, Berkeley, California, United States of America; 6 Department of Medicine, Georgetown University, Washington, District of Columbia, United States of America; Boston University, UNITED STATES

## Abstract

**Background:**

Retention in HIV treatment must be improved to advance the HIV response, but research to characterize gaps in retention has focused on estimates from single time points and population-level averages. These approaches do not assess the engagement patterns of individual patients over time and fail to account for both their dynamic nature and the heterogeneity between patients. We apply group-based trajectory analysis—a special application of latent class analysis to longitudinal data—among new antiretroviral therapy (ART) starters in Zambia to identify groups defined by engagement patterns over time and to assess their association with mortality.

**Methods and findings:**

We analyzed a cohort of HIV-infected adults who newly started ART between August 1, 2013, and February 1, 2015, across 64 clinics in Zambia. We performed group-based multi-trajectory analysis to identify subgroups with distinct trajectories in medication possession ratio (MPR, a validated adherence metric based on pharmacy refill data) over the past 3 months and loss to follow-up (LTFU, >90 days late for last visit) among patients with at least 180 days of observation time. We used multinomial logistic regression to identify baseline factors associated with belonging to particular trajectory groups. We obtained Kaplan–Meier estimates with bootstrapped confidence intervals of the cumulative incidence of mortality stratified by trajectory group and performed adjusted Poisson regression to estimate adjusted incidence rate ratios (aIRRs) for mortality by trajectory group. Inverse probability weights were applied to all analyses to account for updated outcomes ascertained from tracing a random subset of patients lost to follow-up as of July 31, 2015. Overall, 38,879 patients (63.3% female, median age 35 years [IQR 29–41], median enrollment CD4 count 280 cells/μl [IQR 146–431]) were included in our cohort. Analyses revealed 6 trajectory groups among the new ART starters: (1) 28.5% of patients demonstrated consistently high adherence and retention; (2) 22.2% showed early nonadherence but consistent retention; (3) 21.6% showed gradually decreasing adherence and retention; (4) 8.6% showed early LTFU with later reengagement; (5) 8.7% had early LTFU without reengagement; and (6) 10.4% had late LTFU without reengagement. Identified groups exhibited large differences in survival: after adjustment, the “early LTFU with reengagement” group (aIRR 3.4 [95% CI 1.2–9.7], *p =* 0.019), the “early LTFU” group (aIRR 6.4 [95% CI 2.5–16.3], *p <* 0.001), and the “late LTFU” group (aIRR 4.7 [95% CI 2.0–11.3], *p =* 0.001) had higher rates of mortality as compared to the group with consistently high adherence/retention. Limitations of this study include using data observed after baseline to identify trajectory groups and to classify patients into these groups, excluding patients who died or transferred within the first 180 days, and the uncertain generalizability of the data to current care standards.

**Conclusions:**

Among new ART starters in Zambia, we observed 6 patient subgroups that demonstrated distinctive engagement trajectories over time and that were associated with marked differences in the subsequent risk of mortality. Further efforts to develop tailored intervention strategies for different types of engagement behaviors, monitor early engagement to identify higher-risk patients, and better understand the determinants of these heterogeneous behaviors can help improve care delivery and survival in this population.

## Abstract 2

Aaloke Mody and colleagues report engagement trajectories in new ART starters to determine which groups are likely to be lost to follow-up.

## Author summary

**Why was this study done?:**

- Retention in care is dynamic, with patients frequently moving in and out of care, but existing analyses often reduce highly dimensional patient histories into categories of “retained” and “unretained” at a particular time point, thus obscuring diverse patient experiences up until that time.
- Uncovering groups defined by distinctive longitudinal engagement behaviors can both improve identification of patients at elevated risk of bad outcomes and reveal when in follow-up time those vulnerabilities emerge.
- Group-based trajectory modeling is a special case of latent class analysis that groups observations from individuals over time into distinct developmental courses and is a promising, but underutilized, method for characterizing engagement in HIV care.
- We use group-based multi-trajectory modeling to characterize how adherence and retention in care changes over time among HIV-infected patients newly initiating antiretroviral therapy (ART) in Zambia and estimate the association between distinctive patterns of engagement and subsequent mortality.

**What did the researchers do and find?:**

- We used group-based multi-trajectory modeling to identify patient subgroups with distinctive longitudinal patterns with respect to 2 measures of engagement after newly initiating ART: (1) medication possession ratio—a validated adherence metric based on pharmacy refill data—over the past 3 months and (2) and loss to follow-up (LTFU) (i.e., being greater than 90 days late for last visit).
- Among 38,879 patients newly initiated on ART in Zambia, we identified 6 distinct trajectory groups: (1) consistently high adherence and retention (28.5%), (2) early nonadherence but consistent retention (22.2%), (3) gradually decreasing adherence and retention (21.6%), (4) early LTFU with later reengagement (8.6%), (5) early LTFU without reengagement (8.7%), and (6) late LTFU without reengagement (10.4%).
- The longitudinal patterns of engagement behavior that were identified remained very consistent across subpopulations stratified by baseline patient characteristics.
- Sociodemographic, clinical, and facility-level characteristics at the time of ART initiation were not strongly associated with membership in any trajectory group, based on multinomial logistic regression.
- In adjusted multivariable Poisson regression, trajectory group membership was one of the strongest predictors of mortality, with the “early LTFU with reengagement” group, the “early LTFU” group, and the “late LTFU” group having higher rates of mortality than the group with consistently high adherence/retention.

**What do these findings mean?:**

- Patients frequently follow a limited number of distinctive temporal patterns in engagement behaviors that exist across various subpopulations and settings. These patterns may represent generalizable behavioral phenotypes driven by unified sets of underlying behavioral determinants.
- Heterogeneity in longitudinal engagement behaviors is associated with marked differences in the subsequent risk of mortality that are similar in magnitude to, but not captured by, a 500-cells/μl difference in CD4 count at ART initiation.
- As baseline characteristics at the time of ART initiation were poorly associated with trajectory group membership, we believe that longitudinally monitoring early retention behaviors may be the best way to identify high-risk patients and opportunities for targeted intervention.
- Future research should focus on understanding the underlying determinants of these engagement patterns, determining how and when to provide tailored interventions for specific types of engagement behaviors, and developing adaptive treatment strategies that are based on patients’ observed engagement behaviors.

## Introduction

Retention in HIV care is widely suboptimal across sub-Saharan Africa and represents a challenging but modifiable determinant of viral suppression and mortality [1]. Previous studies have highlighted that retention can be dynamic, with patients frequently moving in and out of care [2–7], often for diverse reasons [8,9]. Nevertheless, current analyses often reduce these highly dimensional patient histories into estimates from a single time point and also obscure heterogeneity between patients by estimating only population-level averages [10,11]. These approaches provide little understanding about how individual patient engagement changes over time, how engagement behaviors vary across the population, and, ultimately, how longitudinal differences in retention patterns affect patient outcomes. Deeper exploration of retention by identifying groups of patients with different engagement patterns over time (i.e., distinct trajectories) could reveal uncharacterized but vulnerable populations who require intensified support, as well as the particular periods of time when those vulnerabilities emerge.

Distinct longitudinal patterns of engagement in HIV care can potentially be uncovered using novel analytical approaches such a group-based trajectory analysis and hold great promise for advancing research on novel public health strategies for retention. Such approaches interrogate longitudinal data to identify patient histories defined by patterns over time and thereby uncover “behavioral phenotypes” that may not be captured by existing demographic or clinical characteristics. Characterizing these temporal patterns and phenotypes may help identify high-risk patients and time periods and also may reveal unique opportunities for targeted interventions [8,12–14]. For example, evidence suggests that a large subgroup of antiretroviral therapy (ART) initiators will consistently remain engaged in care and that these patients are likely at low risk for poor outcomes [15]. Thus, their frequency of contact with the formal health system can be safely de-escalated, which is currently the rationale behind many differentiated service delivery models [16–18]. In contrast, some patients may have challenges with visit attendance and adherence but remain in care, while others may oscillate between being retained and not retained. Each pattern presents unique opportunities for when to intervene (i.e., after missed visits or at the time of reengagement in care), and interventions may need to be tailored to the different types of structural, health systems, or psychosocial challenges that underlie the distinctive engagement patterns. Improving our understanding of longitudinal engagement patterns can thus help identify distinct patient phenotypes and inform how to target and prioritize interventions in order to best address the diverse needs of patients [19].

In this analysis, we use group-based multi-trajectory modeling [20–22] to characterize how adherence and retention in care change over time and to identify subgroups that exhibit distinctive patterns of engagement among HIV-infected patients newly initiating ART in Zambia. We further examine baseline patient and facility-level characteristics associated with specific engagement patterns, as well as the association of these engagement patterns with subsequent mortality using population-representative estimates of mortality updated during a large multistage survey sampling study [23].

## Methods

### Ethics statement

The study was approved by the University of Zambia Biomedical Research Ethics Committee and institutional review boards at the University of California, San Francisco, and the University of Alabama at Birmingham School of Medicine. The research in this paper was not prespecified in the original study protocol and consists of secondary analysis of preexisting de-identified data. This paper was prepared according to STROBE guidelines (S1 STROBE checklist).

### Patient population and setting

We analyzed a cohort of HIV-infected adults (greater than or equal to 18 years old) who newly initiated ART between August 1, 2013, and February 1, 2015, at 1 of 64 clinics in Zambia. These clinics were operated by the Zambian Ministry of Health and received technical support from the Centre for Infectious Disease Research in Zambia, a Zambian non-governmental organization that supports implementation of HIV care delivery and research across 4 of the 10 provinces in Zambia. Patients were observed until July 31, 2015. At the time of this study, patients were initially eligible for ART only if they had a CD4 count less than 350 cells/μl, WHO clinical stage 3 or 4, or active tuberculosis, but Zambia then updated its HIV treatment guidelines on April 1, 2014, to expand ART eligibility to those with a CD4 count between 350 and 500 cells/μl as well as to all pregnant and breastfeeding women under Option B+ [24,25]. After initiating treatment, patients were followed up monthly for at least the first 6 months, after which they were eligible to have their visits spaced out to 3-month intervals if considered stable.

### Measurements

Measurements were obtained from the national electronic medical record (EMR) system used in routine HIV care in Zambia, SmartCare. To populate the EMR system, providers first manually complete paper clinical forms during routine patient encounters, and then data clerks enter this information into the electronic database. We used patient sociodemographic characteristics (e.g., age, sex, facility site), clinical characteristics (date of ART initiation, enrollment CD4 count, WHO stage, TB diagnosis), and clinic visit and pharmacy refill history (dates, medications dispensed, next scheduled appointment) for our analyses. Using methods previously described [23,26,27], we measured mortality by updating the existing EMR data with current vital status ascertained after tracing a random sample of patients considered lost to follow-up (defined as being greater than 90 days late to a scheduled appointment or 180 days from any recorded clinic encounter) as of July 31, 2015, and applying inverse probability sampling weights in our analyses.

### Analyses

#### Group-based trajectory model

We used group-based trajectory analysis to identify subgroups that followed distinct longitudinal patterns with respect to engagement over time among patients newly initiating ART. This method assumes that the overall population is made up of distinct, but unobserved (i.e., latent), subpopulations with different behavioral phenotypes and then uses the observed data to estimate both the trajectories of these groups and how they are distributed in the population [20,21]. We used this approach to identify groups of new ART starters who had distinct engagement trajectories with regard to 2 measures of patient engagement. As a metric of treatment adherence, we used the medication possession ratio (MPR) over the past 3 months, which is a validated adherence metric that utilizes pharmacy refill data to calculate the ratio of the number of days a patient has ART in their possession to the total number of days in an interval, and is associated with viral suppression [28–30]. As a metric of retention in care, we used whether patients were in care (i.e., not lost to follow-up) or not. For each individual, time 0 of the analysis was the date of ART initiation, and patients were censored at the time of death, transfer to a new facility, or the end of the observation period (i.e., July 31, 2015). Measures of engagement were repeated at 30-day intervals from the time of ART start until individuals were censored. Of note, patients were not censored at times of loss to follow-up (LTFU) and remained under observation until they met 1 of the 3 censoring criteria (i.e., death, transfer, or database closure). We excluded patients who died or were known to have transferred clinics during the first 180 days on ART since their limited time under observation would have precluded them from developing any meaningful engagement trajectory. Thus, all patients in our analysis had a minimum of 180 days to develop a trajectory prior to being censored.

Statistically, group-based trajectory models use maximum likelihood estimation to estimate both the trajectory of each group (modeled as a function of time using flexible polynomials) and the expected population-level distribution of each group that creates the best fit for the observed data [20,21,31]. In order to identify patient groups that follow joint trajectories with regard to both MPR and retention, we performed a multi-trajectory analysis that simultaneously estimated trajectories for MPR over the past 3 months and in care status [22]. Since the number of groups and the order of the trajectory polynomials (i.e., linear, quadratic, cubic) are not actually known a priori (but must be prespecified when estimating a model), we systematically tested a series of model specifications—first varying the number of groups and then the order of the trajectory polynomials—in order to select the model most optimized for fit and parsimony using the Bayesian information criterion [20–22]. In our analyses, MPR was modeled assuming a censored normal distribution and identity link, and in care status was modeled assuming a binomial distribution and logit link. Using previously validated methods [23,26,27], we incorporated sampling weights based on the inverse of the probability of being selected for tracing in all models in order to account for the updated mortality statuses from tracing a random sample of patients lost to follow-up. Based on this final model—which identifies the group trajectories and their population-level distribution, but not trajectory group membership by individual—we then estimated the probabilities of individuals belonging to a specific trajectory group given their observed engagement patterns (i.e., their posterior probabilities) [20–22,31]. Since not all patients had equal observation times, posterior probabilities were based on comparisons between trajectories and patients’ observed data up until the time of being censored. Individuals were then assigned to the trajectory group to which they most likely belonged based on their estimated posterior probabilities (i.e., maximum probability assignment rule) [32].

Lastly, we assessed the adequacy, fit, and consistency of the trajectories identified in our final model. We assessed adequacy and fit of the final model and group assignment using several established metrics: (1) comparing the proportion assigned to each trajectory group based on posterior probabilities to the estimated distribution of group membership from the initial model, (2) estimating the average posterior probability for individuals assigned to each group using the maximum probability assignment rule, (3) calculating the odds ratio of being assigned to the correct trajectory group when using posterior probabilities as compared to the estimated population-level group distribution, and (4) calculating the entropy statistic, an indicator of separation between trajectory groups [20,21]. Additionally, we performed stratified analyses across strata of sex, age, enrollment CD4 count, facility type (i.e., urban, rural, hospital), and province to assess whether the identified trajectories represented generalizable behavioral patterns that remained consistent across multiple subpopulations.

#### Baseline factors associated with trajectory group membership

First, we characterized the patients in each trajectory group by tabulating baseline patient sociodemographic, clinical, and facility-level characteristics by the group to which individuals were assigned based on their posterior probabilities. Tabulations incorporated the probability weights from sampling that were used in the initial trajectory model. Second, we used multinomial logistic regression to assess for baseline factors that were associated with group membership. We selected covariates using directed acyclic graphs based on a priori hypotheses of causal relationships between baseline characteristics and engagement trajectories. Our model included an interaction between age category and sex and also included a restricted cubic spline of the amount of observation time each patient contributed. Facility-level characteristics included the percentage of all visits at a facility that were scheduled at 90-day (3-month) intervals or greater (as opposed to shorter intervals such as 30 or 60 days) and the mean number of patients seen at the clinic per day. We incorporated probability weights from sampling [23,26,27] and used multiple imputation (*n =* 20) to address missingness in predictor variables (i.e., enrollment CD4 count, enrollment WHO stage, marital status, education status, and HIV disclosure status) [33]. We estimated predictive margins to report the results as the expected distribution of trajectory across baseline characteristics.

#### Mortality by trajectory group membership

Lastly, we sought to assess differences in mortality based on trajectory group membership. We first used the Kaplan–Meier approach to obtain stratified estimates of the cumulative incidence of mortality by trajectory group. Time 0 was date of ART initiation, and patients were administratively censored at the time of transfer or end of observation. We used bootstrapping to obtain 95% confidence intervals. Next, we used Poisson regression with a time offset and robust variances to estimate unadjusted and adjusted incidence rate ratios of mortality by trajectory group. Adjusted models included sociodemographic, clinical, and facility-level characteristics; included a restricted cubic spline of the amount of observation time each patient contributed; and employed multiple imputation (*n =* 20) to address missingness in predictor variables. Both Kaplan–Meier and Poisson regression analyses incorporated probability weights to accommodate updated vital status from tracing patients lost to follow-up. All analyses were conducted with Stata version 15.1 (StataCorp, College Station, TX).

#### Sensitivity analyses

As an individual’s group membership is not actually observed and only predicted based on individuals’ own observed data, we conducted sensitivity analyses (presented in S1 Appendix) using the BCH method to examine the effect of misclassification bias that could arise from the uncertainty in assigning trajectory group membership using the maximum probability assignment rule [32,34]. We report the results using the maximum probability assignment rule as the primary analysis because of the similarity of the results and the increased transparency of this method.

## Results

### Patient characteristics

**Fig 1:**
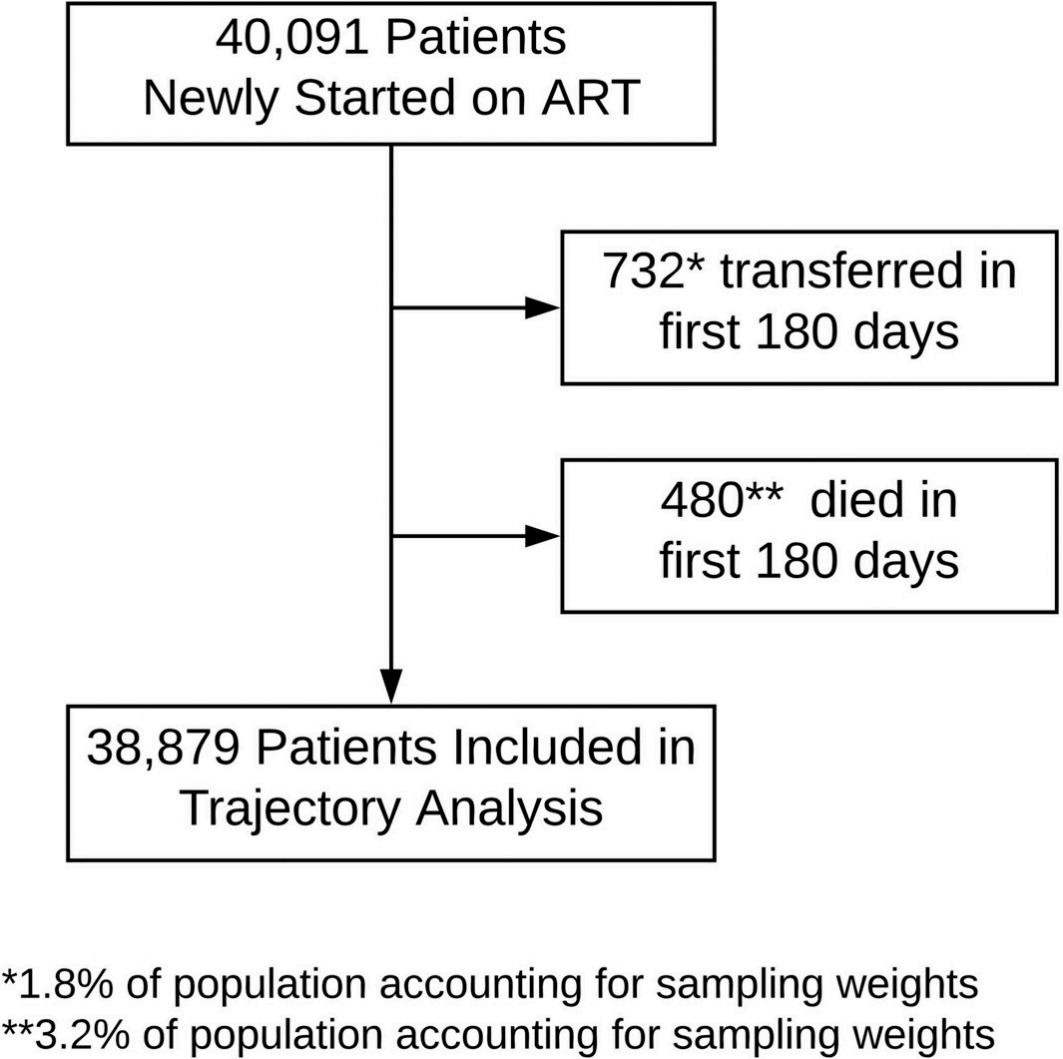
Patient flowchart. Overall, 732 patients were excluded because they transferred care within 180 days of initiating ART, and 480 were excluded because they died within 180 days of initiating ART. After accounting for sampling weights from updated vital statuses after tracing a random sample of patients who were considered lost to follow-up as of July 31, 2015, these excluded patients represented 1.8% and 3.2% of the population, respectively.

Between August 1, 2013, and February 1, 2015, 40,091 patients newly initiated ART at 1 of 64 ART clinics in 4 provinces in Zambia; after accounting for transfers and deaths, 38,879 patients had at least 180 days of observation time and were included in this analysis (Fig 1). Overall, 24,593 patients were female (63.3%), the median age was 35 years (IQR 29–41), the median CD4 count at enrollment into HIV care was 280 cells/μl (IQR 146–431), and the median time from enrollment to ART initiation was 35 days (IQR 14–225) (Table 1). The median duration of observation time per patient was 429 days (IQR 314–571).

**Table 1:** Baseline patient characteristics, *n =* 38,879.

Characteristic	Value
Female sex, *n* (%)	24,593 (63.3%)
Median age, years (IQR)	35 (29–41)
Median CD4 count*, cells/μl (IQR)	280 (146–431)
WHO stage, *n* (%)	
1	18,777 (48.3%)
2	6,645 (17.1%)
3	6,941 (17.9%)
4	607 (1.6%)
Unknown	5,909 (15.2%)
TB in past 6 months, *n* (%)	978 (2.5%)
Median time from enrollment to ART initiation, days (IQR)	35 (14–225)
Marital status, *n* (%)	
Single	3,892 (10.0%)
Married	20,942 (53.9%)
Divorced	4,124 (10.6%)
Widowed	2,730 (7.0%)
Unknown	7,191 (18.5%)
Education, *n* (%)	
None	2,580 (6.6%)
Lower–mid basic	11,538 (29.7%)
Upper basic/secondary	15,804 (40.6%)
College/university	1,479 (3.8%)
Unknown	7,478 (19.2%)
Disclosed HIV status, *n* (%)	
Yes	34,260 (88.1%)
No	1,010 (2.6%)
Unknown	3,609 (9.3%)
Province, *n* (%)	
Lusaka	20,238 (52.1%)
Eastern	7,673 (19.7%)
Southern	5,146 (13.2%)
Western	5,822 (15.0%)
Attends clinic with >50% of visits scheduled at >3 months, *n* (%)	8,453 (21.7%)
Attends clinic with average daily volume ≤ 50 visits/day, *n* (%)	13,327 (34.3%)
Median time observed, days (IQR)	429 (314–571)

*Missing for 7,108 patients.

ART, antiretroviral therapy; IQR, interquartile range; TB, tuberculosis.

### Description of trajectory groups

**Fig 2:**
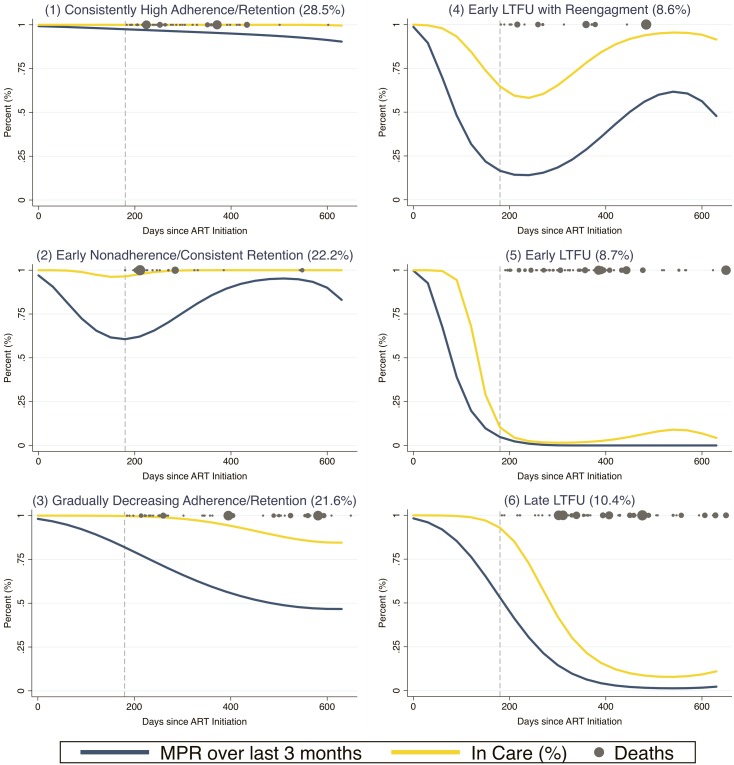
Longitudinal engagement trajectories of new ART starters in Zambia (*n =* 38,879). Group-based trajectory models used sampling weights to account for updated vital statuses after tracing a random sample of patients who were considered lost to follow-up as of July 31, 2015. Grey circles represent deaths and are sized according to sampling weights. All patients included in the model had at least 180 days of observation time, which is represented by the grey dashed line. ART, antiretroviral therapy; LTFU, loss to follow-up; MPR, medication possession ratio.

**Fig 3:**
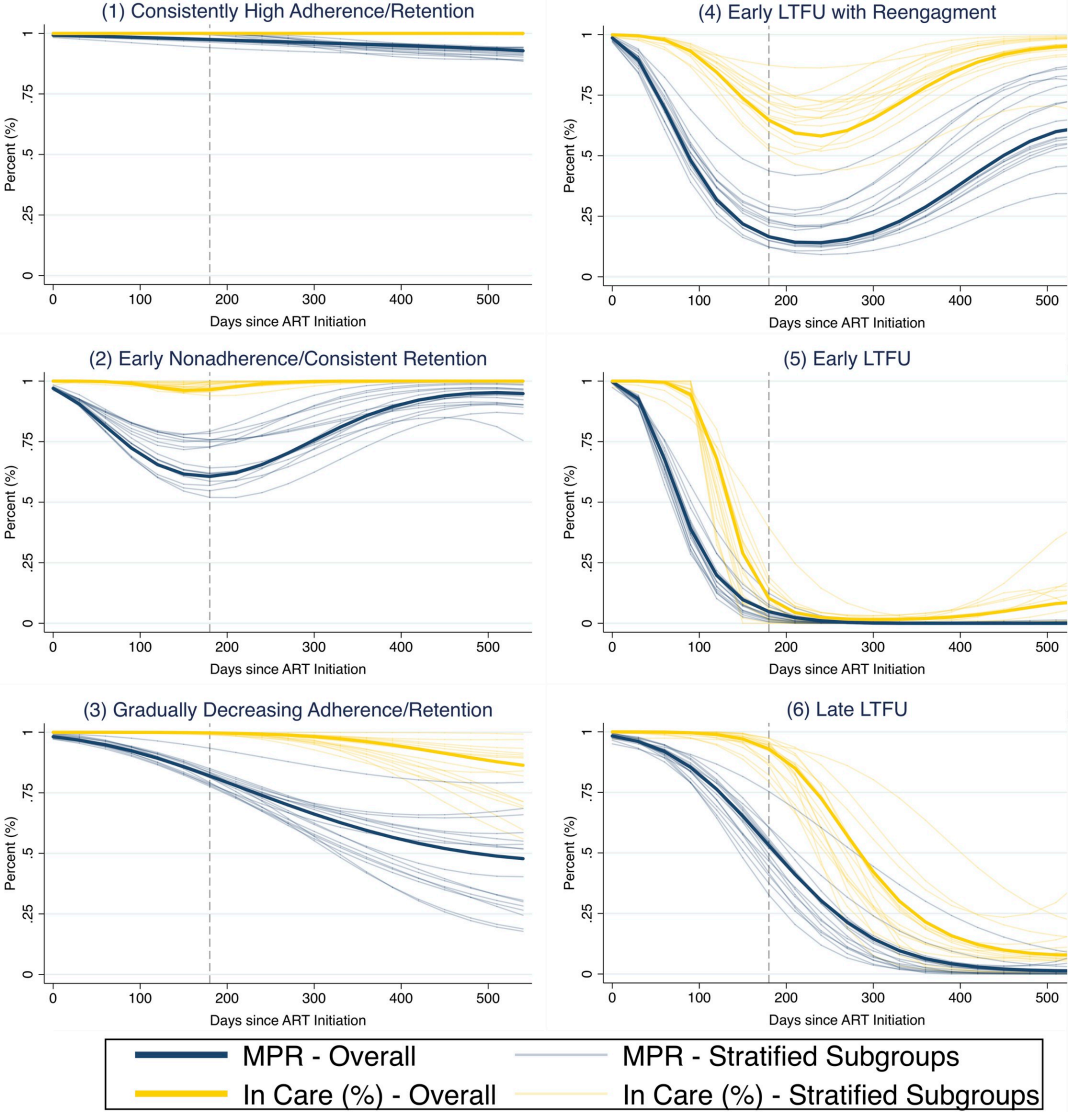
Longitudinal engagement trajectories from analyses stratified across baseline patient characteristics. Stratified analyses were across the strata sex, age, enrollment CD4 count, facility type (i.e., rural, urban, hospital), and province. Group-based trajectory models used sampling weights to account for updated vital statuses after tracing a random sample of patients who were considered lost to follow-up as of July 31, 2015. All patients included in the model had at least 180 days of observation time, which is represented by the grey dashed line. ART, antiretroviral therapy; LTFU, loss to follow-up; MPR, medication possession ratio.

The model with 6 trajectory groups based on longitudinal patterns in adherence and retention was most optimized for fit using the Bayesian information criterion (Fig 2). In this model, the first trajectory group was patients with consistently high adherence and retention (“consistently high adherence/retention” group) and was predicted to account for 28.5% (95% CI 26.7%–30.3%) of the population. The second trajectory group was patients who had suboptimal adherence early who then improved about 1 year after ART initiation, but remained consistently retained in care throughout (“early nonadherence/consistent retention” group, 22.2% [95% CI 19.3%–25.1%] of the population). The third group had gradually decreasing adherence and retention (“gradually decreasing adherence/retention” group, 21.6% [95% CI 19.2%–24.1%] of the population). The fourth group had both poor adherence and poor retention early on, but began to reengage about 1 year after ART initiation (“early LTFU with reengagement” group, 8.6% [95% CI 7.6 to 9.6%] of the population). The fifth group had early nonadherence and LTFU without ever reengaging (“early LTFU” group, 8.7% [95% CI 7.1%–11.9%] of the population). Lastly, the sixth group developed LTFU and poor adherence after initially remaining retained for the first 6 months of treatment (“late LTFU” group, 10.4% [95% CI 8.9%–11.9%] of the population). Table 2 shows diagnostic metrics for our final model and demonstrates that it had very good fit and excellent separation of groups based on well-established metrics. Fig 3 shows that the identified trajectory shapes remained very consistent across subpopulations based on strata of sociodemographic, clinical, and facility characteristics.

**Table 2:** Metrics of adequacy and fit of trajectory model.

Trajectory group	Group average posterior probability	Odds ratio for correct classification	Estimated group distribution based on using maximum probability assignment rule	Estimated group distribution based on initial model	Entropy
Consistently high adherence/retention	0.942	39	0.289	0.285	0.957
Early nonadherence/consistent retention	0.893	29	0.220	0.222	
Gradually decreasing adherence/retention	0.860	22	0.219	0.216	
Early LTFU with reengagement	0.923	128	0.085	0.086	
Early LTFU	0.975	412	0.085	0.087	
Late LTFU	0.918	99	0.101	0.104	

Good model fit indicated by (1) average posterior probability greater than 0.7 for each group, (2) odds ratio of correct classification greater than 5 for each group, (3) close correspondence between the estimated group distribution based on using posterior probabilities and the maximum probability assignment rule and the estimated group distribution from the initial model; and (4) an entropy value greater than 0.8.

LTFU, loss to follow-up.

### Baseline characteristics associated with trajectory group membership

**Fig 4:**
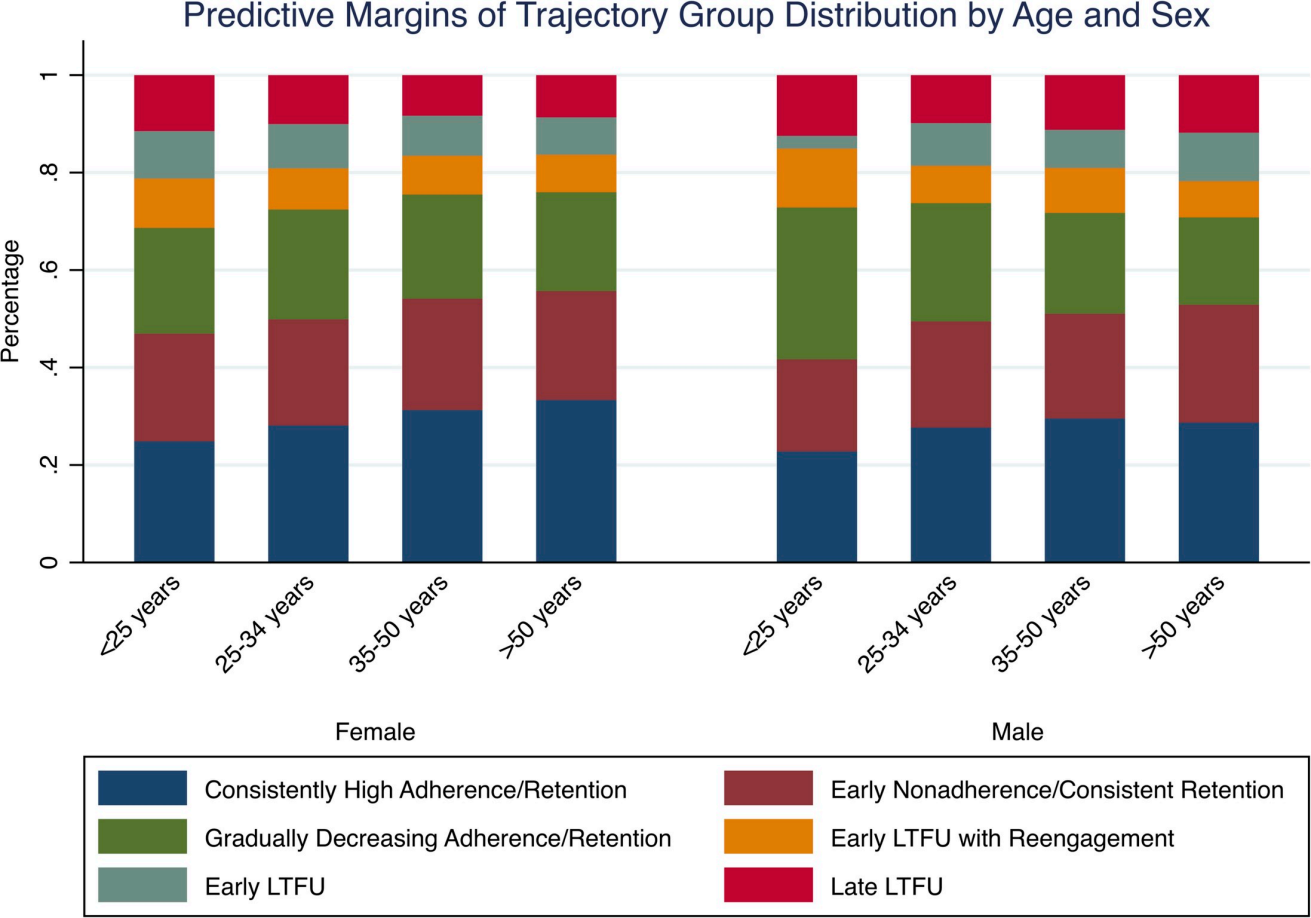
Predictive margins of trajectory group distribution by age and sex (*N =* 38,879). LTFU, loss to follow-up.

Overall, we identified few baseline characteristics that were strongly associated with trajectory group membership (Tables 3 and 4). In multinomial logistic regression, patients who were older, attended a clinic where visits were scheduled at less frequent intervals (i.e., every 90 days as opposed to every 30 days), and were not from Lusaka province were more likely to belong in to the “consistently high adherence/retention” group (Table 4). In addition, patients who were single, had a college/university education, had a CD4 count less than 200 cells/μl, and were from Lusaka province were more likely to be in the “early LTFU” and “late LTFU” groups. Younger females and older males were also somewhat more likely to belong to the “early LTFU” or “late LTFU” group. Patients who were younger and male had a tendency to be in trajectory groups with intermittent engagement (i.e., “gradually decreasing adherence/retention” or “early LTFU with reengagement” group) rather than groups with consistent engagement (i.e., “consistently high adherence/retention” or “early nonadherence/consistent retention” group), but had similar proportions in the trajectory groups with the worst engagement (i.e., “early LTFU” and “late LTFU” groups) (Table 4 and Fig 4). Despite these patterns, however, overall differences across baseline characteristics in the distribution of trajectory groups were small.

**Table 3:** Baseline patient characteristics by trajectory group, *n =* 38,879.

Characteristic	Consistently high adherence/retention (28.5%)	Early nonadherence/ consistent retention (22.2%)	Gradually decreasing adherence/retention (21.6%)	Early LTFU with reengagement (8.6%)	Early LTFU (8.7%)	Late LTFU (10.4%)
Female sex, percent	63.8%	64.1%	62.7%	65.2%	64.1%	58.4%
Median age, years (IQR)	36 (30–43)	35 (29–41)	34 (29–41)	34 (28–39)	34 (29–41)	33 (28–41)
Median CD4 count, cells/μl (IQR)	291 (151–438)	284 (152–431)	286 (158–432)	313 (170–492)	244 (96–445)	254 (122–445)
WHO stage, percent						
1	48.5%	50.1%	49.2%	53.8%	41.6%	48.8%
2	19.7%	16.3%	17.3%	13.3%	15.6%	18.2%
3	15.7%	15.4%	16.7%	16.1%	20.9%	19.2%
4	1.2%	1.5%	1.3%	1.0%	1.4%	1.8%
Unknown	15.0%	16.0%	15.5%	15.8%	20.5%	12.0%
TB in past 6 months, percent	2.1%	2.1%	2.2%	3.1%	2.4%	3.1%
Median time from enrollment to ART initiation, days (IQR)	32 (14–227)	42 (14–285)	41 (15–228)	56 (15–455)	42 (14–190)	44 (14–191)
Marital status, percent						
Single	8.9%	9.2%	10.9%	10.5%	11.3%	21.4%
Married	54.9%	56.3%	55.2%	53.6%	62.2%	51.3%
Divorced	10.6%	10.0%	9.8%	9.3%	9.6%	11.2%
Widowed	8.1%	7.3%	6.3%	5.0%	7.2%	4.9%
Unknown	17.4%	17.2%	17.8%	21.5%	9.7%	11.1%
Education, percent						
None	6.6%	7.3%	5.4%	9.0%	3.7%	6.6%
Lower–mid basic	32.9%	28.0%	30.2%	28.5%	39.0%	22.7%
Upper basic/secondary	39.7%	40.2%	41.2%	37.4%	40.6%	47.3%
College/university	3.6%	4.3%	4.1%	3.6%	6.6%	10.1%
Unknown	17.2%	19.3%	19.1%	21.5%	10.0%	13.4%
Disclosed HIV status, percent						96.0%
Yes	89.4%	88.6%	89.0%	86.7%	86.5%	87.3%
No	2.1%	2.7%	2.1%	1.6%	1.8%	3.7%
Unknown	8.5%	8.7%	8.9%	11.7%	11.8%	9.0%
Province, percent						
Lusaka	39.4%	50.5%	52.4%	68.2%	65.9%	57.1%
Eastern	26.0%	21.4%	17.5%	15.2%	10.2%	12.6%
Southern	20.7%	11.5%	11.1%	5.8%	11.1%	10.7%
Western	13.9%	16.5%	19.0%	10.8%	12.8%	19.6%
Attends clinic with >50% of visits scheduled every 3 months, percent	32.8%	17.8%	19.0%	11.9%	15.4%	22.9%
Attends clinic with average daily volume ≤ 50 visits/day, percent	33.3%	31.7%	36.0%	31.1%	26.0%	32.1%
Median time observed, days (IQR)	415 (303–555)	411 (309–557)	441 (316–577)	438 (322–594)	390 (276–581)	400 (311–514)

All values calculated accounting for sampling weights included in the initial group-based trajectory model.

ART, antiretroviral therapy; IQR, interquartile range; LTFU, loss to follow-up; TB, tuberculosis.

**Table 4:** Predictive margins of trajectory group distribution across baseline patient characteristics from multinomial logistic regression, *n =* 38,879.

Characteristic	Consistently high adherence/retention	Early nonadherence/ consistent retention	Gradually decreasing adherence/retention	Early LTFU with reengagement	Early LTFU	Late LTFU
Overall (from final trajectory model)	28.5% (26.7%–30.3%)	22.2% (19.3%–25.1%)	21.6% (19.2%–24.1%)	8.6% (7.6%–9.6%)	8.7% (7.1%–11.9%)	10.4% (8.9%–11.9%)
Sex and age at enrollment						
Female <25 years old	24.9% (22.7%–27.0%)	22.1% (19.8%–24.4%)	21.7% (18.9%–24.6%)	10.1% (7.4%–12.8%)	9.7% (5.4%–14.1%)	11.5% (7.3%–15.6%)
Female 25–34 years old	28.1% (26.6%–29.6%)	21.8% (20.5%–23.0%)	22.5% (20.8%–24.2%)	8.5% (7.1%–9.8%)	9.1% (6.7%–11.5%)	10.0% (7.7%–12.3%)
Female 35–50 years old	31.3% (29.6%–33.0%)	22.8% (21.4%–24.3%)	21.4% (19.6%–23.2%)	7.9% (6.1%–9.8%)	8.2% (5.5%–11.0%)	8.3% (6.0%–10.6%)
Female >50 years	33.3% (29.5%–37.2%)	22.3% (19.4%–25.2%)	20.3% (17.5%–23.1%)	7.7% (5.4%–10.0%)	7.7% (0.9%–14.4%)	8.7% (3.6%–13.7%)
Male <25 years old	22.8% (16.8%–28.8%)	18.9% (14.0%–23.7%)	31.2% (20.1%–42.3%)	12.1% (3.6%–20.6%)	2.6% (0.0%–6.4%)	12.4% (3.6%–21.3%)
Male 25–34 years old	27.7% (25.2%–30.2%)	21.8% (19.7%–23.8%)	24.3% (21.4%–27.2%)	7.7% (5.9%–9.5%)	8.8% (5.1%–12.4%)	9.8% (6.8%–12.9%)
Male 35–50 years old	29.6% (27.8%–31.3%)	21.5% (19.8%–23.2%)	20.7% (18.9%–22.5%)	9.2% (7.3%–11.2%)	7.8% (5.0%–10.6%)	11.2% (8.2%–14.2%)
Male >50 years	28.7% (25.3%–32.1%)	24.2% (20.7%–27.6%)	17.9% (15.1%–20.7%)	7.4% (5.3%–9.6%)	9.9% (4.4%–15.5%)	11.8% (4.7%–18.9%)
Enrollment CD4 count						
<200 cells/μl	27.6% (26.2%–29.1%)	21.3% (20.0%–22.7%)	21.2% (19.6%–22.8%)	7.5% (6.2%–8.8%)	10.5% (7.9%–13.1%)	11.8% (9.4%–14.2%)
200–350 cells/μl	29.3% (27.6%–30.9%)	23.1% (21.7%–24.5%)	22.6% (21.0%–24.2%)	9.2% (7.4%–11.0%)	5.8% (3.6%–8%)	10.1% (7.7%–12.6%)
351–500 cells/μl	30.8% (29.0%–32.7%)	21.7% (20.1%–23.2%)	24.0% (21.6%–26.4%)	7.7% (6.0%–9.5%)	8.8% (5.9%–11.8%)	6.9% (4.6%–9.1%)
>500 cells/μl	28.6% (26.6%–30.7%)	22.3% (20.4%–24.1%)	19.9% (17.9%–21.8%)	10.4% (7.9%–13.0%)	8.2% (4.5%–11.8%)	10.7% (7.2%–14.1%)
WHO stage						
1	28.3% (27.2%–29.3%)	23.0% (22.0%–23.9%)	22.0% (20.8%–23.2%)	9.3% (8.1%–10.4%)	7.8% (6.1%–9.5%)	9.7% (8.2%–11.3%)
2	30.9% (29.1%–32.8%)	20.4% (18.8%–22.1%)	21.8% (19.8%–23.9%)	7.2% (5.4%–8.9%)	8.9% (5.8%–11.9%)	10.8% (7.8%–13.7%)
3	28.8% (26.8%–30.7%)	20.7% (19.0%–22.5%)	21.9% (20.0%–23.8%)	7.9% (6.4%–9.4%)	10.3% (7.1%–13.5%)	10.4% (7.6%–13.2%)
4	26.0% (20.7%–31.3%)	24.1% (18.4%–29.8%)	22.6% (15.2%–30.1%)	6.5% (3.9%–9.1%)	8.9% (0.0%–18.4%)	11.9% (3.8%–20.0%)
TB in past 6 months						
No	28.9% (28.1%–29.6%)	22.1% (21.4%–22.8%)	22.0% (21.1%–22.8%)	8.5% (7.7%–9.2%)	8.6% (7.3%–9.8%)	10.1% (8.9%–11.3%)
Yes	29.2% (24.5%–33.9%)	20.1% (16.1%–24.1%)	20.4% (16.4%–24.3%)	11.7% (6.3%–17.1%)	6.9% (1.6%–12.2%)	11.7% (3.4%–20.0%)
Time from enrollment to ART initiation						
<14 days	28.6% (27.3%–30.0%)	21.8% (20.4%–23.2%)	21.1% (19.7%–22.6%)	8.5% (7.2%–9.8%)	9.7% (7.4%–12.1%)	10.2% (7.9%–12.5%)
14–90 days	30.7% (29.3%–32.0%)	21.6% (20.5%–22.7%)	22.9% (21.4%–24.5%)	7.1% (6.0%–8.3%)	7.8% (5.8%–9.8%)	9.9% (8.0%–11.8%)
>90 days	27.2% (25.8%–28.5%)	22.6% (21.4%–23.9%)	21.4% (19.9%–22.9%)	10.0% (8.3%–11.6%)	8.5% (6.2%–10.8%)	10.4% (8.1%–12.6%)
Marital status						
Single	26.5% (24.1%–28.9%)	19.5% (17.5%–21.5%)	20.5% (17.6%–23.4%)	9.4% (6.1%–12.7%)	7.9% (4.5%–11.4%)	16.2% (11.6%–20.7%)
Married	28.9% (27.9%–29.9%)	22.5% (21.6%–23.3%)	22.4% (21.2%–23.5%)	8.6% (7.6%–9.6%)	8.9% (7.3%–10.5%)	8.8% (7.4%–10.3%)
Divorced	28.9% (26.8%–31.0%)	22.0% (20.1%–23.8%)	21.8% (19.4%–24.1%)	8.7% (6.5%–10.8%)	7.4% (4.3%–10.5%)	11.3% (7.7%–14.9%)
Widowed	32.5% (29.8%–35.1%)	23.0% (20.6%–25.5%)	21.6% (18.7%–24.5%)	6.6% (4.8%–8.4%)	8.6% (4.0%–13.1%)	7.7% (4.2%–11.2%)
Education						
None	26.7% (24.4%–29.1%)	23.3% (20.9%–25.7%)	23.3% (20.9%–25.7%)	13.1% (9.5%–16.8%)	5.7% (2.8%–8.6%)	10.9% (7.3%–14.4%)
Lower–mid basic	30.1% (28.6%–31.5%)	21.3% (20.1%–22.4%)	21.3% (20.1%–22.4%)	8.7% (7.3%–10.2%)	10.1% (7.7%–12.5%)	7.7% (5.9%–9.5%)
Upper basic/secondary	29.1% (28.0%–30.2%)	22.6% (21.5%–23.6%)	22.6% (21.5%–23.6%)	7.9% (6.8%–8.9%)	7.7% (6.0%–9.3%)	10.6% (8.9%–12.4%)
College/university	23.0% (19.9%–26.1%)	20.8% (17.0%–24.6%)	20.8% (17.0%–24.6%)	7.6% (3.7%–11.6%)	9.6% (4.5%–14.7%)	18.7% (11.7%–25.7%)
Disclosed HIV status						
No	26.1% (21.6%–30.6%)	25.5% (20.0%–31.1%)	19.8% (16.1%–23.6%)	6.3% (2.9%–9.7%)	6.4% (0.5%–13.4%)	15.7% (7.8%–23.6%)
Yes	29.0% (28.2%–29.7%)	21.9% (21.2%–22.6%)	22.0% (21.1%–22.9%)	8.6% (7.8%–9.4%)	8.6% (7.3%–9.8%)	10.0% (8.7%–11.2%)
Province						
Lusaka	23.2% (21.9%–24.4%)	21.4% (20.3%–22.4%)	22.3% (21.0%–23.6%)	11.1% (9.6%–12.5%)	10.5% (8.5%–12.5%)	11.6% (9.5%–13.7%)
Eastern	38.7% (37.3%–40.2%)	23.1% (22.0%–24.3%)	20.3% (19.0%–21.7%)	6.2% (5.4%–6.9%)	4.3% (3.0%–5.6%)	7.3% (5.8%–8.7%)
Southern	36.5% (34.2%–38.9%)	22.3% (20.5%–24.1%)	19.3% (17.4%–21.1%)	5% (3.8%–6.3%)	9.5% (6.2%–12.9%)	7.3% (4.9%–9.7%)
Western	27.5% (25.3%–29.6%)	23.1% (21.2%–25.0%)	25.2% (22.4%–28.1%)	5.8% (4.8%–6.8%)	6.9% (3.8%–10.0%)	11.5% (7.9%–15.2%)
Percent of visits at clinic scheduled at 3 month intervals						
≤50%	25.5% (24.7%–26.3%)	23.4% (22.6%–24.2%)	22.8% (21.8%–23.8%)	9.3% (8.5%–10.1%)	9.3% (7.8%–10.7%)	9.7% (8.4%–11.0%)
>50%	39.9% (37.2%–42.6%)	17.6% (16.1%–19.0%)	19.1% (17.3%–21.0%)	5.4% (3.3%–7.4%)	6.0% (3.5%–8.6%)	12.0% (8.0%–16.0%)
Average daily volume at clinic						
≤50 visits/day	26.5% (25.5%–27.5%)	22.1% (21.1%–23.1%)	24.9% (23.6%–26.2%)	9.1% (8.1%–10.1%)	7.1% (5.7%–8.5%)	10.3% (8.8%–11.7%)
>50 visits/day	30.1% (29.0%–31.2%)	21.9% (21.0%–22.8%)	20.5% (19.3%–21.7%)	8.3% (7.3%–9.3%)	9.2% (7.5%–10.9%)	10.0% (8.3%–11.7%)

Adjusted for restricted cubic splines for the amount of time a patient was under observation.

ART, antiretroviral therapy; LTFU, loss to follow-up; TB, tuberculosis.

### Mortality by trajectory group membership

**Fig 5:**
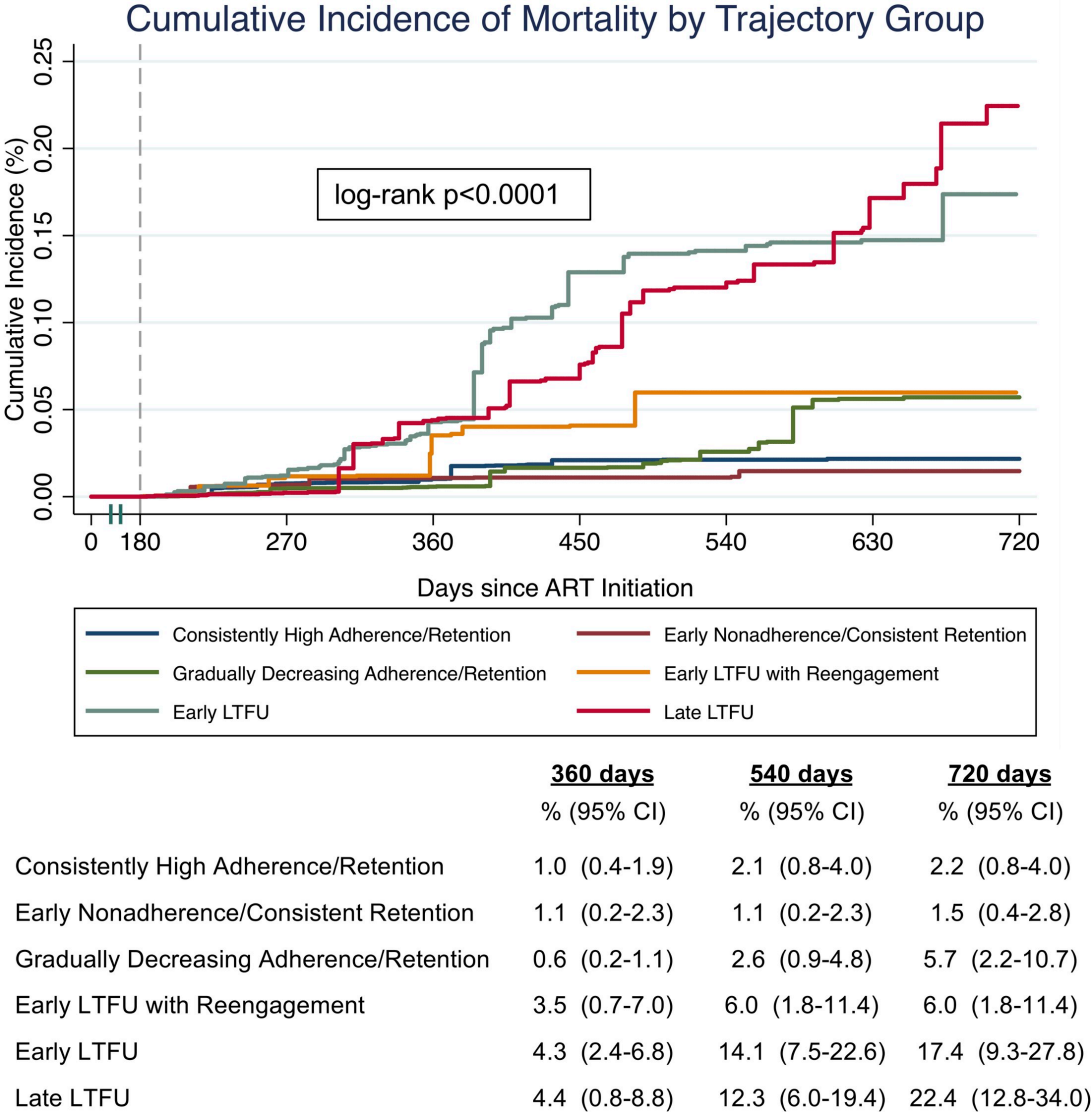
Kaplan–Meier estimates of the cumulative incidence of mortality by trajectory group with bootstrapped 95% confidence intervals (*n =* 38,879). Kaplan–Meier estimates used sampling weights to account for updated vital statuses after tracing a random sample of patients who were considered lost to follow-up as of July 31, 2015. Only patients with at least 180 days of observation time (represented by the grey dashed line) were included in the original group-based trajectory model. ART, antiretroviral therapy; LTFU, loss to follow-up; MPR, medication possession ratio.

There were markedly different risks of mortality based on trajectory group membership (Fig 5). The “early LTFU” group (17.4% [95% CI 9.1%–28.4%]) and “late LTFU” group (22.4% [95% CI 13.4%–34.6%]) had the highest cumulative incidence of mortality at 720 days. The “consistently high adherence/retention” group (2.2% [95% CI 0.8%–4.0%]) and “early nonadherence/consistent retention” group (1.5% [95% CI 0.4%–2.7%]) had the lowest mortality at 720 days, while the “gradually decreasing adherence/retention” group (5.7% [95% CI 2.2%–10.5%]) and the “early LTFU with reengagement” group (6.0% [95% CI 1.8%–11.6%]) had intermediate mortality (Fig 5). In both unadjusted and adjusted Poisson regression, trajectory group membership remained significantly associated with mortality. Even after adjusting for baseline sociodemographic, clinical, and facility-level characteristics, the “early LTFU with reengagement” group (adjusted incidence rate ratio [aIRR] 3.4 [95% CI 1.2–9.7], *p =* 0.019), the “early LTFU” group (aIRR 6.4 [95% CI 2.5–16.3], *p <* 0.001), and the “late LTFU” group (aIRR 4.7 [95% CI 2.0–11.3], *p =* 0.001) had significantly increased rates of mortality as compared to the “consistently high adherence/retention” group (Table 5). Results remained consistent in sensitivity analyses using the BCH method to account for any bias due to misclassification of trajectory group membership (S1 Appendix).

**Table 5:** Poisson regression of the association between trajectory membership and mortality, *n =* 38,879.

Characteristic	Unadjusted IRR (95% CI)	*p-*Value	Adjusted IRR (95% CI)	*p*-Value
Trajectory group				
Consistently high adherence/retention	1.00 (REF)	—	1.00 (REF)	—
Early nonadherence/consistent retention	0.71 (0.23–2.19)	0.55	0.80 (0.27–2.37)	0.69
Gradually decreasing adherence/retention	1.48 (0.55–3.98)	0.44	1.68 (0.67–4.22)	0.27
Early LTFU with reengagement	2.45 (0.84–7.12)	0.099	3.45 (1.23–9.69)	0.019
Early LTFU	6.42 (2.68–15.42)	<0.001	6.44 (2.54–16.29)	<0.001
Late LTFU	5.57 (2.27–13.64)	<0.001	4.71 (1.97–11.28)	0.001
Male sex	—	—	1.15 (0.70–1.88)	0.58
Age, per 10-year increase	—	—	1.36 (1.18–1.58)	<0.001
Enrollment CD4 count, per 100-cells/μl increase	—	—	0.65 (0.52–0.82)	<0.001
WHO stage	—	—		
1			1.00 (REF)	0.44
2			1.71 (0.91–3.20)	
3			1.32 (0.65–2.69)	
4			1.50 (0.43–5.23)	
TB in past 6 months	—	—	0.55 (0.13–2.29)	0.41
Time from enrollment to ART initiation, per 90-day increase	—	—	1.02 (0.97–1.06)	0.47
Marital status	—	—		
Single			0.67 (0.27–1.77)	0.32
Married			1.00 (REF)	
Divorced			1.50 (0.80–2.83)	
Widowed			0.82 (0.41–1.66)	
Education				
None			1.00 (REF)	0.88
Lower–mid basic			0.85 (0.37–1.92)	
Upper basic/secondary			0.72 (0.31–1.68)	
College/university			0.74 (0.20–2.70)	
Disclosed HIV status	—	—	0.57 (0.17–1.86)	0.35
Province	—	—	0.81 (0.46–1.43)	0.47
Lusaka			1.00 (REF)	0.55
Eastern			0.89 (0.49–1.61)	
Southern			0.95 (0.42–2.13)	
Western			1.50 (0.74–3.00)	
Attends clinic with >50% of visits scheduled every 3 months	—	—	1.67 (0.80–3.49)	0.17
Attends clinic with average daily volume ≤ 50 visits/day	—	—	0.84 (0.52–1.35)	0.66

Adjusted model also included restricted cubic splines for the amount of time a patient was under observation.

ART, antiretroviral therapy; IRR, incidence rate ratio; LTFU, loss to follow-up; TB, tuberculosis.

## Discussion

Using group-based trajectory modeling, we characterized 6 trajectory groups among new ART starters in Zambia that followed distinct longitudinal patterns with regard to MPR over the past 3 months and in care status. Based on our model, 28.5% (95% CI 26.7%–30.3%) of patients had consistently high adherence and retention over time (“consistently high adherence/retention” group), 22.2% (95% CI 19.3%–25.1%) had suboptimal adherence early with later recovery but consistent retention (“early nonadherence/consistent retention” group), 21.6% (95% CI 19.2%–24.1%) had gradually decreasing adherence and retention (“gradually decreasing adherence/retention” group), 8.6% (95% CI 7.6%–9.6%) had early nonadherence and LTFU but then had later reengagement (“early LTFU with reengagement” group), 8.7% (95% CI 7.1%–11.9%) had early nonadherence and LTFU without ever reengaging (“early LTFU” group), and 10.4% (95% CI 8.9%–11.9%) had later nonadherence and LTFU without reengaging (“late LTFU” group). The identified trajectory patterns remained consistent across subpopulations stratified by patient characteristics. In multinomial logistic regression, we identified few baseline characteristics strongly associated with trajectory group membership, though patients who were older, attended clinics where visits were scheduled less frequently, and were not from Lusaka Province were slightly more likely to be in the “consistently high adherence/retention” group, and patients who were young females or older males, were single, were college-educated, had low CD4 counts, and were from Lusaka were slightly more likely to be in the “early LTFU” and “late LTFU” groups. Lastly, even after adjusting for well-known predictors of mortality including sex, age, CD4 count, and WHO stage, trajectory group membership (based on longitudinal patterns in adherence and retention) remained one of the strongest predictors of mortality. Compared to those in the “consistently high adherence/retention” group, patients in the “early LTFU with reengagement” group (aIRR 3.4 [95% CI 1.2–9.7], *p =* 0.019), “early LTFU” group (aIRR 6.4 [95% CI 2.5–16.3], *p <* 0.001), and “late LTFU” group (aIRR 4.7 [95% CI 2.0–11.3], *p =* 0.001) all had significantly increased rates of mortality. Overall, these findings provide a comprehensive depiction of engagement over time among new ART starters in Zambia and highlight the importance of this heterogeneity in engagement behavior over time as a critical determinant of patient outcomes.

Characterizing longitudinal patterns of engagement provides a richer and more nuanced understanding of patient adherence and retention as compared to more traditional metrics and can help to identify distinctive behavioral phenotypes. Though previous studies have documented that patients frequently transition in and out of HIV care [2–7], current analyses often reduce the highly dimensional temporal dynamics of retention into cross-sectional summaries such as the proportion lost to follow-up and also obscure heterogeneity between patients behind population-level averages [10,11]. However, as this and other analyses demonstrate, the HIV patient population is frequently composed of heterogeneous groups with different patterns of adherence and retention over time [5,6,19]. For example, some patients remain consistently and optimally engaged in care (i.e., “consistently high adherence/retention” group), some patients are consistently retained but suboptimally engaged (i.e., “early nonadherence/consistent retention” and “gradually decreasing adherence/retention” groups), some patients transition in and out with brief periods of disengagement (“early LTFU with reengagement” group), and some patients are only temporarily engaged in care followed by sustained periods of disengagement (“early LTFU” and “late LTFU” groups). In our analyses, these trajectories remained remarkably consistent across subpopulations that were stratified by varied patient characteristics and care settings. Furthermore, they also comport with previous qualitative work focused on describing different patient journeys and personas [35]. These findings suggest that the trajectories we identified represent more generalizable behavioral phenotypes that could be expected to be observed across a wide range of settings even with more contemporary advances in HIV care. These advances, such as universal testing and treatment, routine viral load monitoring, and differentiated services delivery, may alter the distribution between trajectory groups, but it is unlikely that they would dramatically change the general behavioral patterns underlying each trajectory. Thus, our findings provide a deeper understanding of prototypical engagement behaviors in HIV care over time that remain relevant.

This heterogeneity in the longitudinal engagement behaviors of patients is highly relevant for improving outcomes in public health treatment programs, given that it is highly associated with patient mortality. As compared to the trajectory groups with better engagement (“consistently high adherence/retention” and “early nonadherence/consistent retention” groups), the remaining groups had increased risk of mortality that resembled a dose–response pattern according to how “bad” their engagement trajectory was (i.e., the “early LTFU” and “late LTFU” groups tended to have worse outcomes than the “gradually decreasing adherence/retention” and “early LTFU with reengagement” groups). As even small differences in levels of viremia have been shown to effect subsequent treatment failure and mortality [36–39], this finding is not entirely unexpected, but the degree to which mortality risk is associated with variations in patient behavioral patterns is marked and noteworthy. Indeed, even after adjustment, the difference between the “best” and “worst” engagement patterns (i.e., the behavioral risk) independently translates into the equivalent biological risk associated with an approximately 500-cells/μl decrease in CD4 count. Current strategies to target high-risk patients largely focus on sociodemographic risk factors such as sex, age, and geography at the time of ART initiation, but these do not convey the same degree of mortality risk as the engagement patterns that emerge over time. Furthermore, the mortality gradient across trajectory groups highlights that even smaller lapses in engagement are quite relevant, even though they have garnered less attention than LTFU. Our findings thus highlight the importance of understanding the temporal dynamics of adherence and retention, and of shifting attention towards utilizing this behavioral information to develop intervention strategies tailored to patients’ observed behaviors.

We found few baseline characteristics that were strongly associated with trajectory group membership, and, ultimately, monitoring patients’ actual engagement behaviors over time may be one of the most critical predictors of subsequent risk. Few standard patient characteristics at baseline have proven to be strongly predictive of retention behavior to date, so it is not surprising that we were also unable to find any that meaningfully distinguished between trajectory groups, though, in general, the patterns that we did find do comport with current knowledge of risk factors for poor engagement [40]. Patient-reported barriers to care or unexpected changes in individuals’ lives (referred to as idiosyncratic shocks in the economics literature) have previously been associated with retention, adherence, and even mortality, so more comprehensive and longitudinal patient assessments that include these potential behavioral determinants of engagement would likely further help discriminate trajectory group membership [8,12,41–51]. Ultimately, however, monitoring patients’ actual behavior—which is already readily accessible—and the patterns that emerge over time is likely to provide some of the most useful data for assessing risk for subsequent outcomes and for adapting patients’ treatment plans accordingly. Indeed, in a study from the US that utilized machine learning algorithms to develop a prediction model of retention that incorporated 1,466 variables, patients’ prior history was the most important variable [52]. It is important to note that, in our analysis, patients began differentiating into distinct trajectory groups within the first 180 days even though we included the full amount of observation time to classify patients. Similar to current processes for offering differentiated services delivery, treatment plans could easily be adapted based on these early engagement behaviors as these data are readily available. Future research that could help to refine this strategy includes using machine learning algorithms to incorporate high-dimensional retention data into predictive models and employing sequential multiple assignment randomized study designs (i.e., SMART trials) that leverage patients’ observed behavior or outcomes to assess adaptive treatment strategies.

The fact that engagement patterns remained consistent across patient subpopulations implies that we identified generalizable engagement phenotypes, and this has several important implications for future practice-oriented research agendas. First, the existence of these archetypal engagement patterns suggests that there are potentially unified sets of determinants underlying these different behaviors. Future qualitative research should be undertaken to identify and better characterize these behavioral determinants and to further understand how needs may differ across these groups. Second, the trajectories themselves underscore several time points with important but different opportunities for intervention. For example, the patients at highest risk for mortality do not return after their initial period of engagement. This reveals the need for more intensive and tailored community-based interventions to reach and reengage those who have disengaged (and who are at highest risk), including active tracing, peer navigation, home-based care, and leveraging social networks, particularly since few interventions have been especially successful at preventing LTFU altogether [53]. Furthermore, we found that a group of patients does reengage in care on their own, and the time of reengagement represents another unique opportunity to strengthen engagement—with services such as the Médecins Sans Frontières Welcome Service—among those who have already demonstrated they are at risk for poor engagement [54]. Lastly, to target patients with a history of good retention, differentiated models of care (including multi-month scripting) have recently gained traction as a strategy to reduce the burden of accessing care, with current evidence suggesting improved outcomes [53]. Other strategies that seek to increase the flexibility and convenience of HIV care, such as mHealth interventions, may also be ideal for these patients as well as patients with gradual declines in engagement (who potentially represent a group of patients that want to stay in care but find it difficult to). Future studies that focus on these different behavioral phenotypes could attempt to assess patients’ care preferences and intervention strategies across these specific groups to better understand when, how, and what types of interventions should be targeted toward each type of engagement behavior. Beyond strategies for individual behavioral patterns, our findings also imply that HIV treatment programs will likely need a robust package of diverse types of retention interventions to adequately address the different behavioral phenotypes. Thus, this more nuanced understanding of engagement behaviors paves the way for HIV treatment programs to shift away from what are still frequently one-size-fits-all approaches and to strategically develop more targeted and comprehensive programs to optimize their public health impact.

There are several limitations of our study. First, it is important to note that the trajectory groups that we identified and how patients are then classified into these groups are not necessarily intrinsic properties and only represent systematic attempts to characterize and classify patients based on the available data. This, along with inherent limitations in our data source (such as unequal observation times and measurement error with regard to MPR and retention status), has the potential to lead to classification error or classifications that may not seem intuitive. Nevertheless, model diagnostics indicated a very good fit for the data with clear differentiation between trajectory groups, and results were consistent in sensitivity analyses accounting for any potential misclassification error. Additionally, trajectory patterns remained very consistent across analyses stratified by baseline patient characteristics. This would suggest that, overall, we were successful in both identifying trajectory groups that represent generalizable latent engagement phenotypes and classifying patients into these groups. Second, with latent class methodologies such as group-based trajectory modeling, the observed data are used to define the exposure. That, however, may also introduce bias from survivor effects when using longitudinal data and may limit causal inference, though we did attempt to control for this by adjusting for cubic splines of the amount time each patient was observed. Third, we excluded patients who died or transferred within the first 180 days of treatment in order to ensure that patients had sufficient time under observation to develop a meaningful engagement trajectory. This also precluded us from making any conclusions regarding engagement and mortality in this particularly high-risk early period (i.e., within 180 days of ART initiation), although it is also likely that baseline clinical characteristics such as CD4 count, WHO stage, and presence of opportunistic infections are the primary drivers of mortality in this early period. Fourth, the duration of follow-up was also limited to 2 years, which may have precluded us from assessing more long-term effects of different engagement trajectories such as development of drug resistance. Fifth, we were unable to assess virologic outcomes as viral loads were not routinely collected in Zambia during our study period, though MPR and LTFU are imperfect proxies for virologic outcomes. Lastly, as our data are from 2013 to 2015, their generalizability to current care standards that include universal test and treat, routine viral load monitoring, and differentiated services delivery is uncertain, though our sensitivity analyses do suggest that we were able to identify engagement trajectories that represent more generalizable behavioral patterns that might still be observed even with current care standards.

### Conclusion

In conclusion, we used novel group-based multi-trajectory analysis to identify 6 patient subgroups among patients newly initiating ART in Zambia that followed distinct trajectories with regard to ART adherence and retention in care over time. We found that 19.1% of patients become lost to follow-up within the first year while another 30.2% intermittently have poor adherence and retention. Nevertheless, 50.7% of patients maintain fairly consistent adherence and retention over time. Furthermore, we found that these distinct engagement trajectories were significantly associated with risk of mortality, but we identified few baseline characteristics that strongly predicted subsequent engagement trajectory. These results highlight the importance of capturing these highly dimensional and heterogeneous engagement patterns and using this information to guide research agendas and HIV treatment programs in developing more robust strategies for improving retention. This improved understanding of the heterogeneity in patient behaviors ultimately can be used to effectively and efficiently tailor interventions for a diverse patient population and will be an essential component for implementing more patient-centered HIV care.

## Supporting information

S1 Appendix Supplementary sensitivity analysis to account for misclassification error. (DOCX)

S1 STROBE checklist STROBE checklist. (DOCX)

## Abbreviations

aIRR: adjusted incidence rate ratio
ART: antiretroviral therapy
EMR: electronic medical record
LTFU: loss to follow-up
MPR: medication possession ratio
