# Supplementary material for: Longitudinal engagement trajectories and risk of death among new ART starters in Zambia: A group-based multi-trajectory analysis
Source: PLoS Med. 2019 Oct 29;16(10):e1002959. doi: 10.1371/journal.pmed.1002959 (PMC6818762; doi:10.1371/journal.pmed.1002959)
Supplement: S1 Appendix — (DOCX) [file pmed.1002959.s001.docx]

*S1 Appendix - Sensitivity Analyses using BCH Method to Account for Potential Misclassification Error*

As an individual’s group membership is not actually observed and only predicted based on individuals’ own observed data, we conducted sensitivity analyses to examine the effect of the uncertainty that arises from assigning trajectory group membership using maximum posterior probability assignment rule. Specifically, we used the modified BCH method to account for the uncertainty that arises in group membership, which has been shown to sometimes provide less biased estimates when compared to the maximal probability rule as well as other methods for addressing uncertainty in group membership (i.e., proportionally assigning individuals’ to each trajectory group based on posterior probabilities [proportional assignment method] or randomly imputing trajectory group multiple times based on posterior probabilities and then combining estimates using Rubin’s rule for multiply imputed datasets [multiple pseudo-class draw method]) [1,2]. In the modified BCH approach, patients are assigned to the trajectory group based on the maximal probability rule, but observations are also weighted by the inverse of the expected amount of classification error for this group assignment rule—that is the probability of being assigned to trajectory group X given that one’s “true” trajectory group is in fact Y. We repeated analyses for baseline characteristics associated with trajectory group membership (using multinomial logistic regression), cumulative incidence of mortality at 720 days stratified by trajectory group (using the Kaplan-Meier method with bootstrapped confidence intervals), and the adjusted incidence rate ratio of mortality by trajectory (using Poisson regression) using the modified BCH method.

**Table S1: Predictive Margins of Trajectory Group Distribution Across Baseline Patient Characteristics from Multinomial Logistic Regression using BCH method, n=38,879**

| Predictive Margins of Trajectory Group Distribution Across Baseline Patient Characteristics from Multinomial Logistic Regression using BCH Method, n=38,879 | | | | | | |
| --- | --- | --- | --- | --- | --- | --- |
|  | Consistently high adherence/retention | Early nonadherence/ consistent retention | Gradually decreasing adherence/retention | Early LTFU with reengagement | Early LTFU | Late LTFU |
| Overall (from final trajectory model) | 28.5%  (26.7%-30.3%) | 22.2%  (19.3%-25.1%) | 21.6%  (19.2%-24.1%) | 8.6%  (7.6%-9.6%) | 8.7%  (7.1%-11.9%) | 10.4%  (8.9%-11.9%) |
| Sex and Age at Enrollment |  |  |  |  |  |  |
| Female <25 years old | 24.4%  (22.0%-26.8%) | 21.5%  (19.0%-24%) | 21.4%  (18.1%-24.6%) | 10.5%  (7.6%-13.5%) | 10.0%  (5.4%-14.6%) | 12.2%  (7.5%-16.8%) |
| Female 25-34 years old | 27.6%  (26.0%-29.3%) | 21.3%  (19.9%-22.7%) | 22.2%  (20.3%-24.2%) | 8.8%  (7.3%-10.4%) | 9.4%  (6.8%-11.9%) | 10.6%  (8.1%-13.2%) |
| Female 35-50 years old | 30.8%  (29.0%-32.7%) | 22.4%  (20.7%-24.0%) | 21.1%  (19.1%-23.2%) | 8.3%  (6.3%-10.4%) | 8.5%  (5.6%-11.4%) | 8.9%  (6.2%-11.5%) |
| Female >50 years | 32.8%  (28.7%-37.0%) | 21.9%  (18.6%-25.1%) | 20.1%  (16.9%-23.2%) | 8.1%  (5.5%-10.6%) | 7.9%  (0.7%-15.1%) | 9.2%  (3.5%-15.0%) |
| Male <25 years old | 22.4%  (15.6%-29.2%) | 18.4%  (12.9%-23.9%) | 30.7%  (17.8%-43.7%) | 12.6%  (3.2%-22.0%) | 2.7%  (0.0%-6.7%) | 13.2%  (3.3%-23.0%) |
| Male 25-34 years old | 27.2%  (24.5%-29.9%) | 21.3%  (18.9%-23.6%) | 24.0%  (20.6%-27.3%) | 8.0%  (6.0%-10.0%) | 9.0%  (5.2%-12.9%) | 10.4%  (7.0%-13.8%) |
| Male 35-50 years old | 29.0%  (27.1%-31.0%) | 21.0%  (19.1%-22.9%) | 20.4%  (18.3%-22.5%) | 9.7%  (7.5%-11.8%) | 8.1%  (5.1%-11.0%) | 11.9%  (8.5%-15.3%) |
| Male >50 years | 28.2%  (24.5%-31.9%) | 23.6%  (19.8%-27.4%) | 17.7%  (14.5%-20.8%) | 7.8%  (5.4%-10.2%) | 10.2%  (4.4%-16.1%) | 12.5%  (4.6%-20.5%) |
| Enrollment CD4 Count |  |  |  |  |  |  |
| <200 cells/μL | 27.1%  (25.5%-28.7%) | 20.8%  (19.3%-22.3%) | 20.9%  (19.1%-22.7%) | 7.8%  (6.4%-9.3%) | 10.8%  (8.1%-13.5%) | 12.5%  (9.8%-15.2%) |
| 200-350 cells/μL | 28.8%  (27.1%-30.5%) | 22.6%  (21.0%-24.2%) | 22.3%  (20.5%-24.1%) | 9.6%  (7.6%-11.6%) | 6.0%  (3.6%-8.3%) | 10.8%  (8.0%-13.5%) |
| 351-500 cells/μL | 30.4%  (28.4%-32.4%) | 21.3%  (19.5%-23.0%) | 23.8%  (21.0%-26.5%) | 8.1%  (6.2%-10.0%) | 9.1%  (6.0%-12.3%) | 7.3%  (4.8%-9.8%) |
| >500 cells/μL | 28.1%  (25.9%-30.3%) | 21.7%  (19.7%-23.8%) | 19.6%  (17.4%-21.7%) | 10.9%  (8.1%-13.7%) | 8.4%  (4.6%-12.2%) | 11.3%  (7.5%-15.2%) |
| WHO Stage |  |  |  |  |  |  |
| 1 | 27.8%  (26.6%-28.9%) | 22.5%  (21.4%-23.5%) | 21.7%  (20.3%-23.1%) | 9.7%  (8.5%-10.9%) | 8.0%  (6.2%-9.8%) | 10.3%  (8.6%-12.1%) |
| 2 | 30.4%  (28.4%-32.4%) | 20.0%  (18.2%-21.8%) | 21.5%  (19.2%-23.8%) | 7.5%  (5.5%-9.4%) | 9.1%  (5.9%-12.4%) | 11.5%  (8.1%-14.8%) |
| 3 | 28.3%  (26.2%-30.4%) | 20.2%  (18.3%-22.2%) | 21.5%  (19.4%-23.7%) | 8.3%  (6.6%-9.9%) | 10.6%  (7.2%-13.9%) | 11.1%  (7.9%-14.2%) |
| 4 | 25.5%  (19.8%-31.3%) | 23.6%  (17.3%-29.8%) | 22.3%  (14.2%-30.5%) | 6.8%  (3.9%-9.7%) | 9.1%  (0.0%-19.0%) | 12.6%  (3.5%-21.7%) |
| TB in past 6 months |  |  |  |  |  |  |
| No | 28.4%  (27.5%-29.2%) | 21.6%  (20.8%-22.3%) | 21.7%  (20.6%-22.7%) | 8.9%  (8.0%-9.7%) | 8.8%  (7.5%-10.2%) | 10.7%  (9.3%-12.1%) |
| Yes | 28.6%  (23.5%-33.8%) | 19.6%  (15.1%-24%) | 20.1%  (15.6%-24.6%) | 12.2%  (6.3%-18.2%) | 7.1%  (1.5%-12.7%) | 12.4%  (3.1%-21.7%) |
| Time from enrollment to ART Initiation |  |  |  |  |  |  |
| <14 days | 28.1%  (26.6%-29.6%) | 21.3%  (19.7%-22.8%) | 20.8%  (19.2%-22.5%) | 8.9%  (7.5%-10.3%) | 10.0%  (7.6%-12.5%) | 10.8%  (8.2%-13.4%) |
| 14-90 days | 30.2%  (28.7%-31.7%) | 21.1%  (19.9%-22.3%) | 22.6%  (20.9%-24.4%) | 7.5%  (6.2%-8.8%) | 8.0%  (6.0%-10.1%) | 10.5%  (8.4%-12.7%) |
| >90 days | 26.7%  (25.3%-28.2%) | 22.1%  (20.7%-23.5%) | 21.1%  (19.3%-22.8%) | 10.4%  (8.6%-12.3%) | 8.7%  (6.3%-11.2%) | 11.0%  (8.5%-13.5%) |
| Marital Status |  |  |  |  |  |  |
| Single | 25.9%  (23.3%-28.5%) | 19.0%  (16.8%-21.1%) | 20.1%  (16.9%-23.4%) | 9.8%  (6.2%-13.4%) | 8.1%  (4.5%-11.8%) | 17.1%  (12.0%-22.1%) |
| Married | 28.4%  (27.4%-29.5%) | 22.0%  (21.0%-23.0%) | 22.1%  (20.7%-23.4%) | 9.0%  (7.9%-10.1%) | 9.1%  (7.5%-10.8%) | 9.4%  (7.7%-11.0%) |
| Divorced | 28.4%  (26.1%-30.7%) | 21.5%  (19.4%-23.5%) | 21.4%  (18.9%-24.0%) | 9.1%  (6.7%-11.4%) | 7.7%  (4.4%-10.9%) | 12.0%  (7.9%-16%) |
| Widowed | 32.0%  (29.1%-34.9%) | 22.6%  (19.9%-25.3%) | 21.4%  (18.1%-24.6%) | 6.9%  (5.0%-8.9%) | 8.9%  (4.1%-13.6%) | 8.2%  (4.3%-12.1%) |
| Education |  |  |  |  |  |  |
| None | 26.2%  (23.7%-28.8%) | 22.7%  (20.1%-25.3%) | 19.9%  (16.5%-23.3%) | 13.7%  (9.7%-17.7%) | 5.9%  (2.9%-9.0%) | 11.5%  (7.6%-15.5%) |
| Lower-Mid Basic | 29.6%  (28.1%-31.1%) | 20.8%  (19.6%-22.1%) | 21.9%  (20.2%-23.6%) | 9.1%  (7.5%-10.7%) | 10.4%  (7.9%-12.9%) | 8.2%  (6.2%-10.2%) |
| Upper Basic/Secondary | 28.6%  (27.4%-29.8%) | 22.1%  (20.9%-23.2%) | 21.9%  (20.5%-23.3%) | 8.2%  (7.1%-9.4%) | 7.9%  (6.1%-9.7%) | 11.3%  (9.3%-13.3%) |
| College/University | 22.5%  (19.1%-25.8%) | 20.2%  (16.0%-24.4%) | 19.8%  (14.8%-24.9%) | 8.0%  (3.7%-12.2%) | 9.8%  (4.5%-15.2%) | 19.7%  (12.0%-27.4%) |
| Disclosed HIV Status |  |  |  |  |  |  |
| No | 25.6%  (20.7%-30.5%) | 24.9%  (18.7%-31.1%) | 19.5%  (15.4%-23.7%) | 6.6%  (3.0%-10.3%) | 6.6%  (0.0%-13.9%) | 16.7%  (7.9%-25.6%) |
| Yes | 28.5%  (27.6%-29.3%) | 21.4%  (20.7%-22.2%) | 21.7%  (20.7%-22.7%) | 9.0%  (8.1%-9.9%) | 8.8%  (7.5%-10.2%) | 10.6%  (9.2%-12%) |
| Province |  |  |  |  |  |  |
| Lusaka | 22.7%  (21.3%-24.1%) | 20.8%  (19.7%-22.0%) | 21.9%  (20.4%-23.4%) | 11.5%  (9.9%-13.2%) | 10.8%  (8.6%-12.9%) | 12.3%  (9.9%-14.6%) |
| Eastern | 38.3%  (36.7%-39.9%) | 22.8%  (21.4%-24.1%) | 20.2%  (18.6%-21.8%) | 6.5%  (5.6%-7.3%) | 4.5%  (3.1%-5.9%) | 7.8%  (6.1%-9.5%) |
| Southern | 36.1%  (33.6%-38.6%) | 21.9%  (19.9%-24%) | 19.1%  (17.0%-21.2%) | 5.3%  (3.9%-6.7%) | 9.8%  (6.3%-13.4%) | 7.8%  (5.1%-10.4%) |
| Western | 27%  (24.7%-29.4%) | 22.6%  (20.5%-24.8%) | 24.9%  (21.6%-28.2%) | 6.1%  (5%-7.2%) | 7.1%  (3.8%-10.4%) | 12.3%  (8.2%-16.4%) |
| Percent of Visits at Clinic Scheduled at 3 month intervals |  |  |  |  |  |  |
| ≤50% | 25.1%  (24.2%-26%) | 22.8%  (22.0%-23.7%) | 22.5%  (21.3%-23.6%) | 9.7%  (8.8%-10.7%) | 9.6%  (8.0%-11.1%) | 10.3%  (8.9%-11.7%) |
| >50% | 39.3%  (36.3%-42.3%) | 17.2%  (15.6%-18.8%) | 18.9%  (16.8%-21.0%) | 5.6%  (3.4%-7.9%) | 6.2%  (3.5%-8.9%) | 12.8%  (8.2%-17.3%) |
| Average Daily Volume at Clinic |  |  |  |  |  |  |
| ≤50 visits/day | 26.1%  (25%-27.2%) | 21.6%  (20.5%-22.7%) | 24.5%  (23.0%-26.1%) | 9.5%  (8.5%-10.6%) | 7.3%  (5.9%-8.8%) | 10.9%  (9.3%-12.5%) |
| >50 visits/day | 29.6%  (28.4%-30.8%) | 21.4%  (20.4%-22.4%) | 20.2%  (18.9%-21.6%) | 8.7%  (7.5%-9.8%) | 9.4%  (7.6%-11.2%) | 10.6%  (8.7%-12.6%) |

Footnotes: Adjusted for restricted cubic splines for the amount of time a patient was under observation; ART=antiretroviral therapy; LTFU=loss to follow-up; TB=tuberculosis

**Table S2.** **Association between Trajectory Membership and Mortality using the modified BCH method, n=38,879**

| Association between Trajectory Membership and Mortality using the modified BCH method, n=38,879 | | | |
| --- | --- | --- | --- |
|  | Cumulative Incidence at 720 days^*^  (95% CI) | aIRR^**^  (95% CI) | p-value |
| Consistently high adherence/retention | 2.2 (0.8-4.0) | REF | - |
| Early nonadherence/ consistent retention | 1.5 (0.4-2.7) | 0.80 (0.23–2.78) | 0.73 |
| Gradually decreasing adherence/retention | 5.7 (2.2-10.5) | 1.7 (0.6-4.8) | 0.32 |
| Early LTFU with reengagement | 6.0 (1.8-11.6) | 3.5 (1.1-10.5) | 0.029 |
| Early LTFU | 17.4 (9.1-28.4) | 6.5 (2.4-17.4) | <0.001 |
| Late LTFU | 22.4 (13.4-34.6) | 4.7 (1.8-12.1) | 0.001 |

Footnotes: *Kaplan-Meier Estimates with bootstrapped confidence intervals. **Poisson regression adjusted for sex, age at ART initiation, enrollment CD4, WHO Stage, TB at enrollment, time to ART initiation, marital status, education status, HIV disclosure, province, facility characteristics, and restricted cubic splines for the amount of time a patient was under observation.

References

1. Bray BC, Lanza ST, Tan X. Eliminating Bias in Classify-Analyze Approaches for Latent Class Analysis. Struct Equ Modeling. 2015;22(1):1-11. Epub 2015/01/24. doi: 10.1080/10705511.2014.935265. PubMed PMID: 25614730; PubMed Central PMCID: PMCPMC4299667.
2. Bakk Z, Tekle FB, Vermunt JK. Estimating the Association Between Latent Class Membership and External Variables Using Bias-Adjsuted Three-Step Approaches. Sociological Methodology. 2013;43(1):272–311.
